# Impairment Experiences, Identity and Attitudes Towards Genetic Screening: the Views of People with Spinal Muscular Atrophy

**DOI:** 10.1007/s10897-017-0122-7

**Published:** 2017-06-30

**Authors:** Felicity K. Boardman, Philip J. Young, Frances E. Griffiths

**Affiliations:** 1 Division of Health Sciences, Warwick Medical School, University of Warwick, Coventry, UK; 2 School of Life Sciences, University of Warwick, Coventry, UK

**Keywords:** Spinal muscular atrophy, Genetics screening, Disability, Ethics, Social implications

## Abstract

Developments in genetics are rapidly changing the capacity and scope of screening practices. However, people with genetic conditions have been under-represented in the literature exploring their implications. This mixed methods study explores the attitudes of people with Spinal Muscular Atrophy (SMA) towards three different population-level genetic screening programmes for SMA: pre-conception, prenatal and newborn screening. Drawing on qualitative interviews (*n* = 15) and a survey (*n* = 82), this study demonstrates that more severely affected individuals with early-onset symptoms (Type II SMA), are less likely to support screening and more likely to view SMA positively than those with milder, later onset and/or fluctuating symptoms (Types III/ IV SMA). Indeed, this clinically milder group were more likely to support all forms of screening and view SMA negatively. This paper highlights that screening is a complex issue for people with genetic conditions, and the nature of impairment experiences plays a critical role in shaping attitudes.

## Introduction

As reprogenetic medicine advances, and technologies such as whole genome sequencing and Non-Invasive Prenatal Testing (NIPT) are increasingly becoming part of mainstream NHS healthcare (Genomics England 2012; Wells et al. 2014; UK National Screening Committee 2013), important social and ethical questions emerge around their usage. Chief among these concerns is the question of which conditions the technologies can justifiably be employed to prevent, particularly as the number of genetic disorders that it is possible to detect through these means is rapidly burgeoning (Plantinga et al. 2016; Leo et al. 2016).

People currently living with the genetic conditions that are potential candidates for such population screening have much to contribute to answering these questions. However, their perspectives have been vastly under-explored in the literature (Allyse et al. 2015; Barter et al. 2016; Nuffield Council on Bioethics 2017). Indeed, the literature around expanded genetic screening has instead largely focused on the intended administers and recipients of such screening; the general public (Pei-Jung et al. 2017; Plantinga et al. 2016), new/expectant parents (Norton et al. 2014; Green et al. 1993) and/or health care professionals (Watson et al. 1991). This oversight is striking given the potential for substantial impacts on people with genetic disabilities should screening be introduced. These impacts might include: changes in the public profile of the disease they live with, emotional harm associated with having a condition that wider society seeks to avoid (Boardman 2014; Barter et al. 2016) reduced public funding for biomedical research into treatments for the condition (asscciated with declining numbers of people born with the condition), as well as reductions in the availability of peer and community support (Nuffield Council on Bioethics 2017).

Where the views of adults with genetic disabilities towards screening and testing have been explored, conclusions have been somewhat contradictory, with some studies revealing reticence, ambivalence and even active hostility towards screening (e.g. Barter et al. 2016; Benjamin et al. 1993; Middleton et al. 1998; Stern et al. 2002) and others revealing far more supportive and accepting attitudes (Chen and Schiffman 2000). This strong diversity of views is perhaps unsurprising given the heterogeneity of adults with genetic disabilities, both in terms of the nature of their conditions, but also in terms of the way(s) they are experienced in everyday life. In spite of this, however, the relationship between the nature of a person’s impairment, and their attitudes towards screening has remained under-explored within the literature. Indeed, this has persisted despite the widespread acknowledgement that everyday experiences are critical to understanding the varying attitudes towards treatment and cure amongst different impairment groups (Shakespeare 2006; Bogart 2014; Bogart et al. 2012; Hahn and Belt 2004).

This study, using mixed methods research techniques, addresses this gap in the literature by exploring attitudes towards genetic screening amongst people diagnosed with a condition for which population screening could feasibly soon be offered, Spinal Muscular Atrophy (SMA).

### Spinal Muscular Atrophy (SMA) and Population Level Genetic Screening

Spinal muscular atrophy is an autosomal recessive neuromuscular disorder that has been described as the most common genetic cause of infant death (Butchbatch 2016). As such, it is has been argued that it is a prime candidate for inclusion in expanded genetic screening programmes (Prior 2010). Indeed, the recent FDA approval of nusinersen as a treatment for SMA has re-kindled calls for newborn screening programmes to be introduced in the United States and beyond (Ottesen 2017).

SMA is sub-classified into four main types, based on age of onset and severity, although the boundaries of these classifications are contested (Dubowitz 1991). Type I SMA is the severest form, with onset within the first few months of life and death usually occurring before 18 months. Type II SMA (intermediate) is the most divergent form, with onset usually within the first two years of life. With improved support, lifespan for people with SMA Type II can remain near-normal, although such indviduals remain vulnerable to respiratory infections and complications throughout their lives, which may lead to premature death. Type III SMA is usually diagnosed after the age of 4 years, with the majority of those affected able to sit and stand unaided. Type IV SMA is diagnosed in adulthood, with patients developing generalised muscle weakness. In both Types III and IV there is a gradual deterioration of abilities over time, although life span is usually unaffected (Wang et al. 2007).

All four sub-types are caused by functional loss of the *Survival Motor Neuron1* (*SMN1*) gene, with the clinical severity of the disease believed to be (at least in part) regulated by the copy number of a second *SMN* gene, *SMN2*. As current genetic analysis can detect *SMN1* deletions and assess *SMN2* copy number, they have the potential to accurately sub-type at diagnosis (as confirmed by several small pilot studies in the US). However, as there has been no longitudinal large scale trial, the sensitivity and specificity of a *SMN1 / SMN2* prognostic and diagnostic algorithm is unknown. As a result, there is a wide variety of SMA screening practices in the international arena. In the UK, although prenatal testing and cascade carrier screening are routinely offered to families with a known history of SMA, there is currently no screening programme in place for the general population (UK National Screening Committee 2013; ACOG, 2009; Cartwright 2012). In comparison, some countries have implemented compulsory pre-martial SMA carrier screening programmes (e.g. Qatar) and Israel and Australia offer screening through state-sponsored health care plans (Sukenik-Halevy et al. 2012). In the United States, SMA is currently being considered for inclusion on the state-wide newborn screening panel. It seems reasonable, therefore, to assme that popualtion screening for SMA could one day feasibly be offered within the UK, particularly in the context of emerging therapies.

This study, using mixed methods research techniques, explores attitudes towards genetic screening amongst people diagnosed with SMA. As SMA can present as an adult-onset, childhood onset or congenital impairment (with vastly contrasting levels of severity within these sub-types) a focus on SMA as a candidate genetic screening condition allows an analysis across a broad range of impairment experiences, while maintaining a meaningful comparison between them, within the broad remit of neuromuscular impairment. By so-doing, this study makes an important contribution to an understanding of the views of people with genetic disabilities in an age of expanding genomic medicine.

## Methods

The data reported in this study are derived from a larger study of attitudes towards screening for SMA amongst both adults with SMA and their family members, the findings of which are reported elsewhere (Boardman et al. 2017). While family members are an important stakeholder group in debates around genetic screening in their own right, people with genetic conditions have unique experiential knowledge of the condition in question and are set to affected by population genetic screening in very specific ways (Chen and Schiffman 2000; Allyse et al. 2015). Therefore, the views of this group of adults was considered worthy of a separate and focused analysis. An exploratory sequential mixed methods research design was adopted, and the research took place in three distinct phases.

Within all phases, participants were asked about three potential screening programmes for SMA:

1. A **pre-conception screening programme** (whereby members of the general population are offered screening for their SMA carrier status before conceiving a pregnancy)
2. A **prenatal screening programme** (whereby pregnant women and their partners are offered screening for carriers status, and the foetus tested for SMA where indicated)
3. A **newborn screening programme** (whereby parents of newborn babies are offered genetic screening to determine whether the baby has SMA).

### Phase I: Qualitative Interviews

In-depth qualitative interviews were undertaken with 36 people who either had SMA or had a diagnosis of SMA in their family. These participants were recruited through advertisements placed in the publications of the main support and advocacy group for families living with SMA in the UK, ‘SMA Support UK’. Fifteen interviews were conducted with adults with SMA, and 21 with family members of people with SMA (see Table 1 for breakdown of participants). For the purposes of this study, only the 15 interviews with affected adults were included for analysis. Interviews were designed to explore experiences with SMA, views around/uses of genetic testing technologies and selective termination, as well as perceptions of the possible introduction of population screening for SMA. Participants were eligible for interview if they were aged 18 or over, English speaking and either had SMA themselves, or had at least one diagnosis of SMA in their family. Pregnant women were excluded from the study due to the increased sensitivity of the topic area.

**Table 1:** Qualitative interview participants

Type of SMA	Gender		Age			Total
	Male	Female	18–35	36–50	51+	
Type II SMA	3	5	4	3	1	8
Type III SMA	1	4	0	4	1	5
Type IV SMA	1	1	0	1	1	2
Totals	5	10	5	8	3	15

Data were analysed using Nvivo 10 software by an experienced researcher under the supervision of two senior academics who provided feedback on the developing coding framework. A constructivist approach to grounded theory data analysis was used (Charmaz 2008). This process was inductive, allowing the themes to emerge directly from the data, although unlike traditional grounded theory approaches, the literature was consulted during the data analysis to facilitate refinement. After the initial ‘open coding’ of the data, higher level hierarchical coding was undertaken. A process of coding, refinement of concepts (through data interpretation and consultation with the literature) and re-coding was carried out over a period of five months until ‘saturation’ had occured (i.e. no new concepts were emerging and all of the data were incorporated within the coding framework) (Glaser and Strauss 1967).

### Phase II: SMA Screening Survey (UK)

Following completion of the qualitative analysis, a survey- the SMA Screening Survey (UK)- was developed in order to gauge attitudes across a wider population of families living with SMA. Details of the development of the SMA Screening Survey (UK) have been presented more fully elsewhere (Boardman et al. 2017). In brief, the overarching themes from the qualitative analysis were used to develop both the key domains, but also the individual questions within the survey. Questions that were included to capture demographic information (e.g. religious faith/ethnicity) were either directly replicated from, or appear as modified versions of, questions from the 2011 UK Census survey.

The SMA Screening Survey (UK) contained 22 items. Questions pertaining to views on screening took the form of an attitude statement derived direclty from the qualitative analysis (for example, ‘It would be a loss to society to have less people with SMA coming into the world’) presented in conjunction with a lickert scale. Cognitive interviewing was undertaken with six people with experience of SMA to explore the mental processes that participants used to answer the survey questions (Willis 2005). In addition to the cognitive interviews, the survey was independently reviewed by two expert panels (one professional, one made up of people living with SMA and their families) and the survey questions were further developed in line with their feedback.

Phase II data collection was carried out over a period of ten months, September 2014–June 2015. Two versions of the survey were made available, an online version (hosted on a secure website) and a paper version. The paper version was posted to all members of SMA Support UK (1500 households) in September 2014 and participants were encouraged to distribute it within their networks of friends and family affected by SMA. Potential participants were invited to complete the survey if they were over 18, had not taken part in a Phase I interview and either had SMA themselves, or had at least one diagnosis of SMA in the family.

Demographic variables were stratified as follows: 1) Gender (male (1) v female (0)); 2) Highest qualification (> = degree (1) v < degree (0)); 3) Religious (any) (yes (1) v no (0)); 4) Do you have children (own, fostered or adopted) (yes (1) v no (0)); 5) How do you rate your current health (good (1) v not good (0)); and 6) Are you currently trying to conceive (yes (1) v no (0)). For all questions relating to views on SMA or screening programmes, responses were stratified into two groups: 1) “Agree” which contain strongly agree and agree responses; and 2) “Other” which contained disagree, strongly disagree and neither agree nor disagree responses.

Demographic impacts on responses were assessed using logistic regression analysis; when interpreting the results positive drivers were indicated by an odds ratio > 1; negative drivers were indicated by an odds ratio < 1 (for logistic regression to cut off for significance was set at p < = 0.05). The demographic responses were stratified as binominal variables (as highlighted above) to allow analysis using binominal univariate logistic regression (SPSS software v22, IBM).

The attitudes of adults with SMA on the disease itself and the three proposed screening programmes (pre-conception genetic screening, prenatal screening and newborn screening) were compared to determine if there were any statistical differences. Responses from adults with Types II, III and IV were compared. The individual questions were assessed and then responses correlated against support for screening. For each question the number of “agree’ v “other” responses were reported and statistical differences between distribution of responses for the adults with the different subtypes were assessed using a chi-squared analysis (Graphpad Prism software, v6).

### Phase III: Re-Interrogation of Qualitative Data

Key findings that emerged as significant from the quantitative analysis were explored further within the qualitative data in Phase III of the study. Returning to the qualitative data in an exploratory sequential mixed methods research design has been identified as a research technique particularly useful in drawing out the nuances, complexities and contradictions in participants’ views that would otherwise be missed by monomethod research (Plano Clark and Creswell 2008). Excerpts from the qualitative data were selected for inclusion in this paper if they particularly eloquently communicated or clarified a key finding. All of the qualitative findings reported in this paper are derived from Phase III analysis. Pseudonyms have been used throughout, and all identifying information was removed at the point of transcription in order to safeguard- as far as possible- the anonymity of participants.

## Results

### Quantitative Results

#### Cohort Characteristics

Eighty-two adults with SMA responded to the SMA survey: 27 (33%) with Type II SMA; 31 (38%) with Type III SMA; and 24 (29%) with Type IV SMA. In all, the majority of responders were female (55%), did not have an undergraduate degree (66%), were religious (55%), were parents (either adopted, fostered or their own) (51%), did not rate their current health as good (71%) and were not currently trying to conceive (91%) (Table 2).

**Table 2:** Demographic data for SMA Screening Survey (UK) Participants

Characteristics	Adults with SMA(AwS)					Statistical Comparison*
	All	Type II (*n* = 27)	Type III (*n* = 31)	Type IV (*n* = 24)	Type II v III/IV (*n* = 55)	Type II v III/IV
Gender-no. (%)						0.06
Male	37 (45%)	8 (30%)	14 (45%)	15 (63%)	29 (53%)	
Female	45 (55%)	19 (70%)	17 (55%)	9 (37%)	26 (47%)	
Education						0.0002
Degree or Higher	28 (34%)	17 (63%)	5 (16%)	6 (25%)	11 (20%)	
Other	54 (66%)	10 (37%)	26(84%)	18 (75%)	44 (80%)	
Religious						1.0
Yes	45 (55%)	15 (56%)	17 (55%)	13 (54%)	30 (55%)	
No	37 (45%)	12 (44%)	14 (45%)	11 (46%)	25 (45%)	
Do you have children						0.03
Yes	42 (51%)	9 (33%)	18 (58%)	15 (63%)	33 (60%)	
No	40 (49%)	18 (67%)	13 (42%)	9 (37%)	22 (40%)	
How would you rate you current health						0.6
Good	24 (29%)	9 (33%)	10 (32%)	5 (21%)	15 (27%)	
Other	58 (71%)	18 (67%)	21 (68%)	19 (79%)	40 (73%)	
Are you and parent currently trying to get pregnant						0.21
Yes	7 (9%)	4 (15%)	1 (3%)	2 (8%)	3 (5%)	
No	75 (91%)	23 (85%)	30 (97%)	22 (92%)	52 (95%)	

Logistic regression analysis was performed to determine if there was a difference in any of the stratified demographic variables for the survey questions. Significant differences were associated with gender and highlighted that male responders appear to have more negative views of SMA (Table 3). Using the female responders as the reference, logistic regression suggests that males with SMA did not think people with SMA could live fulfilling lives (OR: 0.26; *p* = 0.02; Table 3); they also thought SMA causes people to suffer (OR 3.89 l *p* = 0.004; Table 3) and that it would not be a loss to society if fewer people with SMA were being born (OR 0.38; *p* = 0.04; Table 3).

**Table 3:** Comparison of the views of adults with SMA on the impact of SMA

	Adults with SMA					Statistical Comparison
	Adults with SMA (all; *n* = 82)	Type II (*n* = 27)	Type III (*n* = 31)	Type IV (*n* = 24)	Type II v III/IV (*n* = 55)	Type II v III/IV
Question						*P* Value
People with SMA can live a fullfilling life						0.04
Agree	65 (79%)	25 (93%)	24 (77%)	16 (67%)	40 (80%)	
Other	17 (21%)	2 (7%)	17 (23%)	8 (33%)	15 (20%)	
Having SMA causes people to suffer						0.001
Agree	43 (52%)	7 (26%)	20 (65%)	16 (67%)	36 (65%)	
Other	39 (48%)	20 (74%)	11 (35%)	8 (33%)	19 (35%)	
People with SMA have heightened Intelligence						<0.0001
Agree	33 (40%)	20 (74%)	11 (35%)	2 (8%)	13 (24%)	
Other	49 (60%)	7 (26%)	20 (65%)	22 (92%)	42 (74%)	
People with SMA are well supported by society						0.51
Agree	12 (15%)	5 (19%)	5 (16%)	2 (8%)	7 (13%)	
Other	70 (85%)	22 (81%)	26 (84%)	22 (92%)	48 (87%)	
Quality if life varies across the different type of SMA						1.0
Agree	74 (90%)	25 (93%)	29 (94%)	20 (83%)	49 (89%)	
Other	8 (10%)	2 (7%)	2 (6%)	4 (17%)	6 (11%)	

We have previously shown that adults with Type II have a more positive view on the disease than adults with Type III (Boardman et al. 2017; Table 4). Similar differences to those reported between Types II and III were also seen between adults with Type II and IV SMA (Table 4). More Type II than Type IV adults thought that people with SMA could live fulfilling lives (93% v 67%, respectively; *p* = 0.01; Table 4). Conversely, fewer Type II than Type IV adults agreed that SMA causes people to suffer (26% v 67%, respectively; *p* = 0.005; Table 4). For both of these questions, there were no significant difference between adults with Type III and IV (Table 4). However, the most interesting differences were associated with perceived heightened intelligence in people with SMA, for which there seemed to be an inverse relationship between the clinical severity of disease the responders had, and their agreement that SMA causes heightened intelligence (Type II: 74%, Type III: 35%, Type IV: 8%; Table 4).

**Table 4:** Views of Adults with SMA on Pre-Conception Genetic Screening (PCGS)

	Adults with SMA					Statistical Comparison
	Adults with SMA (all; *n* = 82)	Type II (*n* = 27)	Type III (*n* = 31)	Type IV (*n* = 24)	Type III/IV (*n* = 55)	Type II v Type III/IV
Question						*P* Value
Identifying SMA carries before pregnancy would affect people’s choice of reproductive partners						1.0
Agree	43 (52%)	14 (52%)	19 (61%)	10 (42%)	29 (53%)	
Other	39 (48%)	13 (48%)	12 (39%)	14 (58%)	26 (47%)	
Identifying SMA carries in the general population will lead to carriers feeling stigmatised						0.47
Agree	34 (41%)	13 (48%)	10 (32%)	11 (46%)	21 (38%)	
Other	48 (59%)	14 (52%)	21 (68%)	13 (54%)	34 (62%)	
Identifying SMA carries before pregnancy will reduce the number of SMA-associated terminations						0.07
Agree	57 (70%)	15 (56%)	25 (81%)	17 (71%)	42 (76%)	
Other	25 (30%)	12 (44%)	6 (19%)	7 (29%)	13 (24%)	
Identifying SMA carries in the general population will increase awareness of SMA as a condition						0.11
Agree	69 (84%)	20 (74%)	29 (94%)	20 (83%)	49 (89%)	
Other	13 (26%)	7 (26%)	2 (6%)	4 (17%)	6 (11%)	
Pre-conception screening is a form of social engineering						0.16
Agree	36 (44%)	15 (56%)	9 (29%)	12 (50%)	12 (38%)	
Other	46 (56%)	12 (44%)	22 (71%)	12 (50%)	34 (625%)	
I would support a pre-conceptoin genetic screen for SMA						0.44
Agree	57 (70%)	17 (63%)	27 (87%)	13 (54%)	40 (73%)	
Other	25 (30%)	10 (37%)	4 (13%)	11 (46%)	15 (27%)	

#### Attitudes Towards pre-Conception Genetic Screening

While 70% of the combined cohort supported pre-conception genetic screening, only 54% of Type IV adults were in favour of this form of screening (Table 5); this was lower than both Type II adults (63%) and Type III adults (87%); the difference between the Type III and Type IV adults was significant (*p* = 0.006; Table 5), highlighting diagnosis of SMA in adulthood may impact the view screening.

**Table 5:** Views of Adults with SMA on Prenatal Genetic Screening (PNGS)

	Adults with SMA (all; *n* = 82)	Type II (*n* = 27)	Type III (*n* = 31)	Type IV (*n* = 24)	Type III/IV (*n* = 55)	Type II v Type III/IV
Question						*P* Value
Identifying SMA in pregnancy would lead to fewer people with SMA being born who could live fulfilling						0.33
Agree	51 (62%)	19 (70%)	18 (58%)	14 (58%)	32 (58%)	
Other	31 (38%)	8 (30%)	13 (42%)	10 (42%)	23 (42%)	
Screening for SMA in pregnancy would enable everyone to make informed decisions						0.24
Agree	65 (79%)	19 (70%)	26 (84%)	20 (83%)	46 (84%)	
Other	17 (21%)	8 (30%)	5 (16%)	4 (17%)	9 (16%)	
Screening for SMA in pregnancy will prevent unnecessary suffering						0.009
Agree	35 (43%)	6 (22%)	17 (55%)	12 (50%)	29 (53%)	
Other	47 (57%)	21 (78%)	14 (45%)	12 (5%)	26 (47%)	
Screening for SMA in pregnancy will raise awareness of the condition in the general population						0.13
Agree	66 (80%)	19 (70%)	28 (90%)	19 (79%)	47 (85%)	
Other	16 (20%)	8 (30%)	3 (10%)	5 (21%)	8 (15%)	
It would be a loss to society to have fewer people with SMA coming into the world						0.0006
Agree	32 (39%)	18 (67%)	8 (26%)	6 (25%)	14 (25%)	
Other	50 (61%)	9 (33%)	23 (74%)	18 (75%)	41 (75%)	
It would be difficult for pregnant couples to refuse screening for SMA during pregnancy						0.79
Agree	25 (30%)	9 (33%)	7 (23%)	9 (38%)	16 (29%)	
Other	57 (70%)	18 (67%)	24 (77%)	15 (62$)	39 (71%)	
Screening for SMA in pregnancy is useful even if the type of SMA can bot be determined						0.01
Agree	52 (63%)	12 (44%)	22 (71%)	18 (75%)	40 (73%)	
Other	30 (37%)	15 (56%)	9 (29%)	6 (25%)	15 (27%)	
Termination of milder forms of SMA is unfortunately necessary to reduce the number of children with severe SMA being born						0.09
Agree	19 (23%)	3 (11%)	10 (32%)	6 (25%)	16 (29%)	
Other	63 (77%)	24 (89%)	21 (68%)	18 (75%)	39 (71%)	
I would support a prenatal screening programme for SMA						0.02
Agree	57 (70%)	14 (52%)	25 (81%)	25 (75%)	43 (78%)	
Other	25 (30%)	13 (48%)	6 (19%)	6 (25%)	12 (22%)	

The only other statistical differences between the differed sub-types have previously reported (Type II v III; Table 5). Interestingly, the majority Type IV adults: 1) did not think identifying carriers would alter their choice of reproductive partners (58%- this figure was higher than for Type II (48%) and Type III (39%) adults, although the differences were not significant; Table 5); 2) did not think carriers would be stigmatised; and 3) did believe it would reduce the number of terminations; did believe it would increase SMA awareness in the general population. The lack of significance between the Type II v Type IV and Type III v Type IV for the majority of questions, when there is a significant difference between the Type II v Type III, highlights that the reviews of the Type IV patients are moderate- falling (in general) between those held by the other Types.

#### Attitudes Towards Prenatal Screening

Comparing the support for prenatal screening highlighted no significant differences between Type IV adults and the other two forms (Type II v IV: 52% v 75%, *p* = 0.08; Type III v IV: 81% v 75%, *p* = 0.61; Table 6). Compared to the other sub-groups, more Type IV adults supported prenatal screening that both pre-conception screening (Table 5) and newborn screening (Table 7).

**Table 6:** Views of Adults with SMA on Newborn Genetic screening (NGS)

	Adults with SMA					Statistical Comparison
	Adults with SMA (all; *n* = 82)	Type II (*n* = 27)	Type III (*n* = 31)	Type IV (*n* = 24)	Type III/IV (*n* = 55)	Type II v III/IV
Question						*P* Value
Identifying SMA at birth would lead to better support for children and families						0.17
Agree	76 (93%)	27 (100%)	27 (87%)	22 (30%)	49 (89%)	
Other	6 (7%)	0 (0%)	4 (13%)	2 (8%)	6 (11%)	
Identifying SMA at birth would extend the life expectancy of SMA children						0.004
Agree	41 (50%)	20 (74%)	14 (45%)	7 (29%)	21 (50%)	
Other	41 (50%)	7 (26%)	17 (55%)	17 (71%)	34 (62%)	
Identifying SMA at birth and not during pregnancy removes parents’ ability to make informed decisions about bringing SMA children into the world						0.48
Agree	39 (48%)	11 (41%)	16 (52%)	12 (50%)	28 (51%)	
Other	43 (52%)	16 (59%)	15 (48%)	12 (50%)	27 (49%)	
Identifying SMA before symptoms emerge will prevent families and children enjoying life while they are symptom free						0.46
Agree	26 (32%)	7 (26%)	9 (29%)	10 (42%)	19 (35%)	
Other	56 (68%)	20 (74%)	22 (71%)	14 (58%)	36 (65%)	
Identifying SMA at birth will help research by enabling more children to enrolled into clinical trials early on						1.0
Agree	63 (77%)	21 (78%)	21 (68%)	21 (88%)	42 (76%)	
Other	19 (23%)	6 (22%)	10 (32%)	3 (12%)	13 (24%)	
Identification of SMA at birth would interfere with the early bonding process						0.51
Agree	12 (15%)	5 (19%)	4 (13%)	3 (12%)	7 (13%)	
Other	70 (85%)	22 (81%)	27 (87%)	21 (88%)	48 (87%)	
Identification of SMA at birth would the diagnosis easier for parents to accept						0.47
Agree	36 (44%)	10 (37%)	15 (48%)	11 (46%)	26 (47%)	
Other	46 (56%)	17 (63%)	16 (52%)	13 (54%)	29 (53%)	
Identifying SMA at birth would spare the difficulties associated with finding a diagnosis for a child later on						0.42
Agree	61 (74%)	22 (81%)	22 (71%)	17 (71%)	39 (56%)	
Other	21 (26%)	5 (19%)	9 (29%)	7 (29%)	16 (29%)	
Identifying SMA at birth is important, even if the type can not be determined						0.57
Agree	64 (78%)	20 (74%)	25 (81%)	19 (79%)	44 (80%)	
Other	18 (22%)	7 (26%)	6 (19%)	5 (21%)	11 (20%)	
Identifying SMA at birth is important because it will enable parents to make informed decisions about future pregnancies						1.0
Agree	67 (82%)	22 (81%)	26 (84%)	19 (79%)	45 (82%)	
Other	15 (18%)	5 (19%)	5 (16%)	5 (21%)	10 (18%)	
It is unthical to screen newborns for conditions that have no effective treatment						1.0
Agree	8 (10%)	3 (11%)	2 (6%)	3 (12%)	5 (9%)	
Other	74 (90%)	24 (89%)	29 (94%)	21 (88%)	50 (91%)	
I would support a Newborn screening programme for SMA						0.78
Agree	61 (74%)	21 (78%)	24 (77%)	16 (67%)	40 (73%)	
Other	21 (26%)	6 (22%)	7 (23%)	8 (33%)	15 (27%)

**Table 7:** Views of Adults with SMA on Newborn Genetic Screening (NGS)

		Adults with SMA			Statistical Comparison		
	Adults with SMA (all; *n* = 82)	Type II (*n* **= 27)**	Type III (*n* = 31)	Type IV (*n* = 24)	Type II v Type III	Type II v Type IV	Type III v Type IV
**Question**					*P* Value	*P* Value	*P* Value
Identifying SMA at birth would lead to better support for children and families					0.05	0.12	0.59
Agree	76 (93%)	27 (100%)	27 (87%)	22 (92%)			
Other	6 (7%)	0 (0%)	4 (13%)	2 (8%)			
Identifying SMA at birth would extend life expectancy of SMA children					0.02	0.001	0.22
Agree	41 (50%)	20 (74%)	14 (45%)	7 (29%)			
Other	41 (50%)	7 (26%)	17 (55%)	17 (71%)			
Identifying SMA at birth and not during pregnancy removes parents ability to make informed decisions about bringing SMA children into the world					0.41	0.51	0.91
Agree	39 (48%)	11 (41%)	16 (52%)	12 (50%)			
Other	43 (52%)	16 (59%)	15 (48%)	12 (50%)			
Identifying SMA before symptoms emerge will prevent families and children enjoying life while they are symptom free					0.79	0.23	0.32
Agree	26 (32%)	7 (26%)	9 (29%)	10 (42%)			
Other	56 (68%)	20 (74%)	22 (71%)	14 (58%)			
Identifying SMA at birth will help research by enabling more children to be enrolled into clinical trials early on					0.39	0.36	0.08
Agree	63 (77%)	21 (78%)	21 (68%)	21 (88%)			
Other	19 (23%)	6 (22%)	10 (32%)	3 (12%)			
Identification of SMA at birth would interfere with the early bonding process					0.55	0.55	0.96
Agree	12 (15%)	5 (19%)	4 (13%)	3 (12%)			
Other	70 (85%)	22 (81%)	27 (87%)	21 (88%)			
Identification of SMA at birth would make the diagnosis easier for parents to accept					0.38	0.52	0.85
Agree	36 (44%)	10 (37%)	15 (48%)	11 (46%)			
Other	46 (56%)	17 (63%)	16 (52%)	13 (54%)			
Identifying SMA at birth would spare the difficulties associated with finding a diagnosis for a child later on					0.35	0.37	0.99
Agree	61 (74%)	22 (81%)	22 (71%)	17 (71%)			
Other	21 (26%)	5 (19%)	9 (29%)	7 (29%)			

Compared with Type II adults, a larger percentage of Type IV adults thought prenatal screening would prevent suffering (55% v 22%, *p* = 0.01; Table 6) and that screening was important even if the type could not be determined (71% v 44%, *p* = 0.02; Table 6). There were also fewer Type IV adults who thought that it would be a loss to society if fewer people with SMA were born (26% v 67%, *p* = 0.002; Table 6). In comparison, there was no significant difference between responses from Type III and Type IV adults for any of the prenatal questions. This demonstrates that the Type II adults appear to have the most negative views on prenatal screening, which is underscored by their positive views of the disease and its impact. These positive views do not appear to be widely held in the adults living with the milder forms of the disease.

#### Attitudes Towards Newborn Screening

The majority of responders were in favour of newborn screening (74%; Table 7), with no significant difference between the levels of support in Type II adults (78%), Type III adults (77%) and Type IV adults (67%) (Table 7). When the responses were assessed together, there was more support among adults with SMA for newborn screening than either pre-conception or prenatal screening (74% v 70% v 70%; Table 4, 5, 6 and 7). The only significant difference related to whether newborn screening would extend the life expectancy of affected children: significantly more Type II adults thought this was the case compared with Type IV adults (74% v 29%, respectively; *p* = 0.001; Table 7). In all other questions, there were good levels of agreement; adults from all sub-types believed newborn screening would allow early enrolment on clinical trials, would mitigate the effects of a late diagnosis and would enable all parents to make informed decisions about subsequent pregnancies (Table 7). Importantly, all groups agreed that diagnosis at birth was important, even if the type of SMA could not be determined (Table 7). This highlighted a significant difference in attitudes to prenatal and newborn screening in adults with Type II (Tables 6 and 7). This suggests that for adults with Type II SMA, issues around selective pregnancy termination are the most troubling aspect of SMA screening.

### Qualitative Results

In total, 15 adults with SMA participated in an in-depth interview (see Table 1). Their experiences with SMA as well as their reproductive decisions and attitudes were explored.

#### SMA Type and Views on Screening: ‘I Just don’t see SMA as a big Deal’

Screening for SMA emerged from the qualitative analysis as a divisive topic, which elicited a range of viewpoints. As highlighted by the quantitative findings, adults diagnosed with Type II SMA were more critical of screening than their counterparts with Types III and IV. This finding was also reflected in the qualitative dataset. All but one of the eight participants with Type II SMA expressed significant concerns about what screening would mean for adults like themselves currently living with SMA, as well as broader concerns about the implications that a permissive attitude towards genetic screening would have for other disabled people and wider society.

Catalina has Type II SMA, is 35 years old, living independently (assisted by support workers) and works part-time. She was diagnosed with SMA at the age of 18 months and, despite initially learning to walk, became a fulltime wheelchair user at the age of two. Catalina was very clear in her views that genetic screening was a practice that she could not support:

Oh god yeah, I just hate the idea of it [genetic screening], hate it. I mean it’s like genocide for the modern era isn’t it? It’s portrayed as this sophisticated and progressive new thing, this wonderful development and ‘isn’t it great that we have all this new technology?’, but in reality all they’re doing is bumping the babies off, aren’t they? You know, what’s progressive about that?...[…]…I’m very concerned about it, yes, because it shows you really what this society thinks about disabled people.

[Catalina, 35, Type II]

For adults with Type II SMA who were living fulfilling, independent and productive lives, it is not difficult to see how screening did not align with their own personal goals, which often focused on physical and attitudinal barrier removal and equal participation in society. Indeed, perceptions of SMA- and what sort of life was perceived possible for a person diagnosed with SMA- were critical to understanding screening attitudes across all types and experiences of SMA.

Amy is in her early thirties and, like Catalina, was diagnosed with SMA Type II at an early age (28 months), coming to rely on a wheelchair for mobility from the age of three. At the time of interview, Amy was working full time and living with her husband. When asked about her perceptions of both SMA and screening, Amy responded in the following way:

You see I don’t agree with any sort of screening for SMA, but I guess that’s because I just don’t see SMA as a big deal. I mean….well ok, yes, it’s a big deal- I can’t walk and I have a lot of weakness in my arms that limits me in some ways, but other than that, it hasn’t held me back at all. I don’t think my life would have turned out much different if I hadn’t had it, if you see what I mean. So I suppose I just…I just think there are far worse things you can have in life than SMA, you know? […]… I have a friend who’s ill with depression, she’s had it for years, and to me, she struggles with that so much more than I do. But I don’t think we’d be having this conversation about screening for depression would we? I don’t think it would be seen as acceptable to screen out depressives, would it? [laughs] So yeah… I just don’t really get it.

[Amy, 35, Type II]

Amy’s contrast between depression, which she terms an ‘illness’ and her SMA, which she accepts as an integral aspect of her being (and not one that has limited her life) was a striking theme across her interview. For Amy, her non-support of screening was entirely grounded in her view of SMA, which, she argues, is not a ‘big deal’.

De Wolfe (2002) in her attempt to demarcate the blurry boundaries between ‘illness’ and ‘disability’, as they emerge in the disability rights and medical sociology literature, has argued that a key difference between the two concepts lies in the accommodation of suffering. While, for political ends, disability rights supporters have traditionally been eager to separate out the experiential reality of suffering from definitions of impairment and disability, De Wolfe (2002) argues that such a severance overlooks the needs and rights of the chronically ill, for many of whom ‘illness’ necessarily, and by definition, causes suffering (p. 225).

This presentation of SMA as a ‘disability’ as opposed to an ‘illness’ was in stark contrast to the accounts of adults with Types III and IV SMA, who, as a group, expressed considerably more ambivalence about the reality of living with SMA than adults with Type II.

Virginia is in her late 40s and was diagnosed with Type IV SMA at the age of 30. While she believes the onset of her symptoms was triggered by the birth of her child (Paul) at the age of 22, it took 8 years (and many misdiagnoses) to finally have her diagnosis confirmed. Now at the age of 48, Virginia has given up full time work due to fatigue and is only able to walk short distances. Her son, Paul, now 26, has not undergone testing for SMA and is not displaying symptoms. He undertakes regular care work for his mother following the breakdown of her marriage. Virginia described her views in the following way:

Well, for me, when I had Paul, I didn’t even realise that I had SMA, so I didn’t have any screening for him, but it’s something I feel very strongly about now…. that if I could go back and do it all again [pregnancy] I would absolutely [screen]. I think it’s really important to know what you’re getting into, and for me, SMA is a big no-no. I’m not saying I have a terrible life, but it’s definitely gone downhill since I got the SMA. It had a major part to play in the breakdown of my relationship with Paul’s father. I also used to work in [shop] fulltime, but it just got to the point that I couldn’t be on my feet for long periods, and I was devastated to lose that job. And you know, it isolates you, not working, and not being able to get out and about like you used to. So if I’d known I had this condition, there’s no way I’d have risked Paul getting it. To be fine up to a point then go downhill, it’s cruel really, and not something I’d want for my child.

[Virginia, 48, Type IV]

Informed by her experiences of SMA, Virginia’s sense of ‘genetic responsibility’ (Kenen 1994) was clearly expressed in her reaction to screening technologies. In contrast to Amy- and in spite of having a milder clinical diagnosis than Amy- life with SMA was, for Virginia, punctuated by periods of major loss and decline and instilled in her a sense of moral obligation to prevent transmission to her offspring. Indeed, shame, guilt and blame for a parents’ genetic endowment have been widely observed within the literature in relation to heritable conditons (Dragonas 2001; Hallowell et al. 2006; Reed 2009) and the majority of parents who participated in this study reported some degree of sensed culpability for the condition in their family. Virginia’s negative experiences of SMA heightened her sense of obligation. Indeed, she was already married, a parent and in a physically demanding job by the time her muscle weakness onset, a situation which she stated both caused her considerable anxiety (regarding the potential transmission of the condition to her son) as well as the eventual loss of her much-loved career and deterioration of her marriage. Unlike Amy, who was able to make life choices around her disability from the outset, the unanticipated onset of a serious neuromuscular condition meant a significant re-formulation of Virginia’s day-to-day life.

Such experiences were not uncommon amongst other people diagnosed with later onset forms of SMA; all but one of whom described experiencing significant losses (career, parenting experiences, significant relationships, hobbies) after the onset of their condition. Locock et al. (2009), using Bury’s (1982) theory of ‘biographical disruption’, have argued that people living with degenerative neuromuscular disease must engage in continual cycles of ‘biographical disruption’ (as their disease progresses and they lose function), followed by periods of ‘biographical repair’, during which they attempt to adjust to their new circumstances and abilities. References to these cycles of crisis and then re-adjustment also appeared across the accounts of adults with later onset forms of SMA and has also been noted in the limited literature on the experiences of adults with Type III SMA (Lamb and Peden 2008; Ho et al. 2016). For Virginia, SMA was experienced as a disruptive intrusion into her life, and one that required her to make continual adjustments. This conceptualisation logically led her to view SMA as something that she would want to prevent occurring in her child’s life.

#### SMA, Identity and Screening: ‘I Want My Life- Just without the SMA’

For many adults with late onset forms of SMA, the changes brought about through the onset of their condition not only had a significant impact on the circumstances of their daily lives, but also on their sense of personal identity. Ryan was in his fifties at the time of his interview, having been diagnosed with Type IV SMA in his early thirties. Prior to the onset of his condition, Ryan had worked as a martial arts coach. While still involved with the martial arts and fitness industry, Ryan described his disappointment at recently having to take on less physically demanding roles. While still able to walk at the time of interview, Ryan described now using walking aids (crutches) for longer distances and sometimes a wheelchair. Ryan described his experiences in the following way:

To be honest, the biggest impact [of SMA Type IV] for me has been in terms of how I feel about myself- how I see myself. My muscle mass has decreased significantly since then [onset of SMA], and when I go to the gym now I can’t look at myself in the mirror because I just….I don’t recognise who I see…[…]….I had to give [colleague] a lift home the other day and he saw my crutches in the car, and I felt mortified. Mortified that this is who I’ve become. He was fine about it, but I find it very hard to admit…that I’ve got a disability.

[Ryan, 54, Type IV]

It has long been argued that men’s experiences with disability are at odds with social norms of masculinity with its emphasis on ‘virility, autonomy and independence’ (Asch and Fine 1988). However, as Shuttleworth et al. (2012) highlight, proponents of this argument overlook the various points of intersectionality between impairment experiences and masculinities. Indeed, for Ryan, the onset of SMA forced him to reconcile his new disabled identity, ‘this is who I’ve become now’ with his identity as a previously able-bodied and fit man.

Being able to separate out SMA from one’s sense of personal identity appeared particularly critical to understanding differences in screening attitudes amongst adults with different types of SMA. While adults with Type II spoke of SMA as constituting part of their very being, ‘it’s just who I am’ (Amy), adults with later onset forms of SMA (Types III and IV) were more likely to view SMA as external to their sense of self, which in turn impacted their views of screening. Ryan went on to describe his views of screening for SMA in the following way:

You see, I see screening as completely necessary because I don’t hold much faith in being able to cure it, so the only way to beat this thing is through screening. I know you will get some who will say ‘yes but you can still have a reasonable life with this condition’, and yes you can, but life shouldn’t be about just ‘managing’ should it? Just getting by? You know, I manage, I get by, but a life without this condition would have been preferable. And to me, that’s the point. That’s why we need screening to happen, I want my life- just without SMA.

[Ryan, 54, Type IV]

As Ryan’s account exemplifies, viewing SMA as external to one’s sense of self enabled a conceptualisation of screening as a process capable of eradicating a disease, rather than a particular sub-group of people who have that disease. This dislocation between sense of self and SMA emerged strongly in the accounts of those adults who supported screening, and yet was resolutely absent for participants like Catalina, for whom screening was likened to genocide, designed to eradicate not the incidence of a condition, but a particular ‘kind’ of person.

## Discussion

This study is the first to describe and compare the views of adults with different types of SMA (II-IV) towards three potential population level screening programmes for SMA (pre-conception, prenatal and newborn). The analysis has underscored significant differences between adults with Type II SMA and their counterparts with SMA Types III and IV, a finding which has been alluded to in other studies of adults with SMA (e.g. Kruitwagen-van Reenen et al. 2016; Jeppesen et al. 2010). Indeed, despite participants with Type II experiencing the severest clinical presentation and earliest onset of SMA represented within this sample, adults with Type II reported far more positive views of the condition than those with milder presentations. More severely affected participants were more likely to report high quality of life, to refute the claim that SMA necessarily involves suffering and were more likely than their less severely affected counterparts to see positive attributes associated with SMA, such as heightened intelligence (Von Gontard et al. 2002).

Given this relative positivity displayed by adults with Type II SMA, it is perhaps unsurprising that, as a group, they were more supportive of newborn screening (which would not alter the number of children being born with SMA) compared to prenatal screening (which would potentially increase the number of SMA-related terminations). Indeed, the qualitative and quantitative data both provided clear evidence that the lower levels of support for screening amongst people with Type II SMA stemmed from a fundamental conviction that life with SMA is of considerable value, and consequently that it would be of detriment to have less people with SMA being born. However, it is important to bear in mind when interpreting these data that in spite of this conviction, most still agreed that a pre-conception (63%) or prenatal programme (52%) should be available to the general population, even if they might not make use of genetic technologies within their own reproductive decisions.

These findings are supported by the (somewhat limited) literature exploring the views of affected families and individuals towards population-level genetic screening, where such screening is broadly supported in spite of ambivalence towards their usage (Maxwell et al. 2011; Skinner et al. 2003).

Jeppesen et al. (2010) have argued that due to the early onset of Type II, families with affected children are able to access childhood disability services which are typically more substantial in their provision than those available for disabled adults (Campbell et al. 2016). Early supportive environments have been described as critical to the installation of an enduring positive self-image amongst children with disabilities (Hauser-Cram et al. 2001). Adults with Type II SMA who were interviewed for this study emphasised the importance of these early positive experiences. In particular, the critical role of parents in supporting the transition to adulthood was emphasised. The introduction of the Equality Act (2010) and the protection of the rights of disabled people to work, have children and otherwise participate in society has also meant that opportunities for adults with all types of SMA have improved considerably in recent years. Many such adults are now entering higher education, living independently (through the use of self-directed care plans), undertaking paid employment and becoming parents themselves (Jeppesen et al. 2010). In spite of this, however, most adults with SMA Type II (81%) reported feeling unsupported by wider society, suggesting that their positivity about SMA cannot solely be attributed to improved social and environmental arrangements. Rather, it appeared to be inextricably bound up with how they viewed and experienced their impairment and how they incorporated it within their lives.

Adults affected by the clinically milder forms of SMA (Types III and IV) were more likely to hold negative views of the condition and to more strongly support the forms of screening with the potential to reduce the number of births of SMA children (pre-conception and prenatal genetic screening). Adults with these forms of SMA typically have normal gross motor development prior to the onset of symptoms, which in some instances may not start until late middle-age. Kruitwagen-Van Reenen et al. (2016) have argued that discrepancies between self-reported quality of life across SMA types are due primarily to the delayed onset of the condition in its milder forms. Becoming disabled in early-mid adulthood is a very different experience to being born with the condition, with the onset of the condition invariably involving a re-calibration of an individual’s hopes, dreams and expectations of their life (Locock et al. 2009).

Writing on the concept of cure, Tom Shakespeare (2006) has argued that different ‘impairment groups’ have highly contrasting views on the possible amelioration of their condition, depending, largely, on the nature of their experience with that condition. Those people living with relatively fixed and/or congenital impairments, he argues, are typically more accepting and well-adjusted to them than people whose conditions onset later in life, or whose symptoms fluctuate and/or deteriorate (Shakespeare 2006: 106). This argument is supported by studies within the psychological and rehabilitation literature, where similar differences in attitudes have been observed between people born with their condition and those who acquire it (for example through traumatic spinal cord injury) with the latter group more fervently pursuing treatments and cure (e.g. Bogart 2014; Bogart et al. 2012; Hahn and Belt 2004).

It has been postulated that personal identification with the condition, and the adoption of a ‘disabled identity’ (Watson 2002) is critical to understanding this phenomenon. Indeed, people born with their impairments are more likely to view it as an integral aspect of their personhood (having always been there), which in turn, invariably impacts their views towards its ammeloriation, whether this be through cure or through genetic screening programmes (Kruitwagen-van Reenen et al. 2016). For those who strongly identified with their impairment, it is not difficult to see how the practice of screening could be interpreted as a negative evaluation of their own lives (Sinason 1992). As Edwards (2004) has argued, the ‘expressivist objection’, that is, the hurt and offence that many disabled feel towards the practices of prenatal testing and selective pregnancy termination (Parens and Asch 2000), only makes sense if the disability is considered to be ‘identity constituting’ in some way. Indeed, similar objections are typically not made in relation to other areas of preventative medicine, such as childhood vaccinations (Malek 2010).

Differences in the degree of personal identification with SMA was also evident in the data relating to health. It is noteworthy that when compared to adults with Type II, participants with Types III or IV were more likely to rate their health as poor. This finding might be considered surprising given that people with Type II SMA are (clinically) more severely affected by the disease; they are more likely to suffer chest infections and respiratory complications, are more likely to need nutritional support and surgical interventions for orthopaedic complications (such as Scoliosis and join contractures) than people diagnosed with SMA Types III or IV (Wang et al. 2007). However, this finding can be explained by the observation that adults with Type II SMA separate out their (relatively static and ever-present) disability from their understanding of health and illness, a phenomenon which has been referred to as ‘response shift’ (Kruitwagen-van Reenen et al. 2016: 5). As adults with Types III and IV SMA are likely to have spent a large proportion of their lives symptom-free, the SMA-onset is more likely to be experienced as a ‘threat’ to their health and wellbeing (Shakespeare 2006: 107), rather than entirely separate from it, leading to perceptions of SMA as an ‘illness’ rather than disability, and consequently as something in need of correction or treatment (Boardman 2013).

In conclusion, this study has revealed that the type of SMA affecting a person, and consequently the nature of their experiences with that impairment, has the greatest influence on genetic screening (non)support than any other factor (age, gender, educational background, religion etc.), with support for genetic screening declining the more severely affected a person is. It has been argued that personal identification with, and acceptance of a disabled identity (which is in turn associated with an earlier onset of the condition and a relatively static disease trajectory) is key to understanding this finding. While this concept has previously been explored in relation to the notion of cure (Shakespeare 2006; Hahn and Belt 2004), this study underscores the need to transfer this analysis and understanding of the experiential realities of the lives of disabled people to the arena of selective reproduction. As genomic medicine advances, ability to detect (and consequently screen for) genetic conditions is now far outstripping ability to treat and cure them, rendering the perspectives of people set to be directly affected by this expanded screening (people with genetic disabilities themselves) ever more important in determining, and implementing, the screening agenda.

### Practice Implications

This study emphasises the need of genetic counselors to be attentive to the experiential dimensions of impairment and personal identification with genetic disease in counseling contexts. Disabled people identify more or less with their impairment for a range of reasons, and age of onset/severity are key components of this relationship that may have serious implications for reproductive attitudes. People with disabilities have long been understood as having a fraught relationship with genetic medicine more broadly (e.g. Catalina within this study), with the association with eugenics posing particular challenges (Peterson 2012). Identity politics are key to understanding these tensions, and this study highlights the need for open dialogue between genetic counselors and disabled patients surrounding the nature, meaning and significance of their impairment experiences and how these relate to decisions to use, or not use, genetic technologies.

As capacity to offer screening for ever-larger numbers of rare genetic conditions expands, people living directly with these conditions have an increasing role to play in the concomitant decisions around which conditions should, and which should not, be included on such expanded screening panels. Representing the ‘best experts’ (Petersen 2006) on their own conditions, it is critical that policy makers, clinicians and scientists both value, and make use of, the experiential knowledge of disabled people to inform such decisions. Indeed, as this study highlights, reliance on clinical disease severity to determine a condition’s suitability for inclusion on genetic screening panels (e.g. Leo et al. 2016) may not adequately target those conditions which have the largest negative impact on a person’s day-to-day life.

Indeed, disabled people’s experiences are also relevant to the micro-level decisions made by members of the general population undergoing screening (Shakespeare 2005). Involving people currently living with the screened-for conditions in the education and training of the clinicians who will deliver screening (for example involving them in the production of patient literature and facilitating contact between prospective parents and those living with the screened-for conditons as appropriate) is a key means through which the experiential knowledge of disabled people and their families can be utilised and valued in screening contexts. Different online methods for conveying these insights have been developed (Ahmed et al. 2007; Telling Stories 2007), however, further research is warranted to explore the most effective and appropriate means of delivering this information in the context of high yield genetic screens.

### Research Recommendations

Further research is warranted to explore how adults with conditions that have contrasting presentations to SMA (e.g. those involving chronic pain or behavioural/cognitive symptoms), those that are treatable (e.g. Haemophilia) or those which are early onset, but that which can nevertheless significantly fluctuate or deteriorate from the outset (e.g. Cystic Fibrosis, Duchenne Muscular Dystrophy) view the possibility of genetic screening. Research is also required to explore the quantity, nature and preferred mode of delivery of information about the genetic conditions that can now be screened for within the general population. While some of these concerns are currently being addressed within studies exploring consent processes for additional findings in genomic sequencing studies (e.g. Cornelis et al. 2016), whether and how information needs alter and fluctuate over the course of reproductive decision-making has yet to be thoroughly explored.

### Study Limitations

Due to confidentiality and data protection issues, no identifiable data were asked of individuals who participated in the SMA Screening Survey (UK), including IP addresses (where the survey was completed online). This meant that there was no mechanism in place to prevent an individual completing multiple surveys. Moreover, there was no way of verifying that the participant fitted the inclusion criteria to participate in the survey. Participants were furthermore accessed through a national support group, personal networks and a patient registry rather than neuromuscular clinics, which may have introduced bias. Due to the very poor prognoses associated with Type I SMA, the adults with SMA who participated in this study were largely affected with clinically milder forms of SMA which invariably will have altered their percetpions of the disease. However, we feel that this sample bias does not negate the value of the perspectives of more mildly affected adults, and indeed, even in its milder forms, SMA is still a condition with significant implications for those who live with it.

A further potential source of bias within the sample relates to parental status, with a higher number of parents within the Type III and IV groups than Type II. This is in spite of the increasing number of peope affected by Type II SMA becoming parents overall (Pugh et al. 2000). Parental status might have influenced perceptions of screening amongst these more mildly affected adults as they were more likely to have previously considered the possibility of SMA in their own child

## References

[CR1] Ahmed S, Bryant L, Hewison J (2007). Balance is in the eye of the beholder: Providing information to support informed choices in antenatal screening via antenatal screening web resource. Health Expectations.

[CR2] Allyse M, Minear M, Berson E, Sridhar S, Rote M, Hung A, Chandrasekhara S (2015). Non-invasive prenatal testing: A review of international implementation and challenges. International Journal of Women’s Health.

[CR3] American Congress of Obstetricians and Gynaecologists. (2009). Spinal Muscular Atrophy: ACOG Committee Opinion, 432, http://www.acog.org/Resources-And-Publications/Committee-Opinions/Committee-on-Genetics/Spinal-Muscular-Atrophy

[CR4] Asch A, Fine M, Fine M, Asch A (1988). Introduction: Beyond pedestals. Women with disabilities: Essays in psychology, culture and politics.

[CR5] Barter, B., Hastings, R. P., Williams, R., Huws, J. C. (2016). Perceptions and discourses relating to genetic testing: Interviews with people with down syndrome. *Journal of applied research in intellectual disabilities, 30*(2), 1–12. 10.1111/jar.12256 27168113

[CR6] Benjamin C, Colley A, Donnai D, Kingston H, Harris R, Kerzin-Storrar L (1993). Neurofibromatosis type 1 (NF1): Knowledge, experience, and reproductive decisions of affected patients and families. The Journal of Gene Medicine.

[CR7] Boardman, F. (2013). Screening dilemmas: Disease, disability or something in-between? *Bionews, 732* http://www.bionews.org.uk/page_365114.asp.

[CR8] Boardman, F. K. (2014) The expressivist objection to prenatal testing: The experiences of families living with genetic disease. *Social Science & Medicine, 107*, 18–25 10.1016/j.socscimed.2014.02.025 24602967

[CR9] Boardman F, Young P, Griffiths F (2017). Population screening for spinal muscular atrophy: A mixed methods study of the views of affected families. American Journal of Medical Genetics Part A.

[CR10] Bogart K (2014). The role of disability self-concept in adaptation to congenital or acquired disability. Rehabilitation Psychology.

[CR11] Bogart K, Tickle-Degne L, Nalini A (2012). Compensatory expressive behavior for facial paralysis: Adaptation to congenital or acquired disability. Rehabilitation Psychology.

[CR12] Bury M (1982). Chronic illness as biographical disruption. Sociology of Health & Illness.

[CR13] Butchbatch M (2016). Copy number variations in the *Survival Motor Neuron* genes: Implications for spinal muscular atrophy and other neurodegenerative diseases, Frontiers in molecular biosciences.

[CR14] Campbell F, Biggs K, Aldiss S, O’Neill P, Clowes M, McDonagh J, While A, Gibson F (2016). Transition of care for adolescents from paediatric services to adult health services. Cochrane Database of Systematic Reviews.

[CR15] Cartwright, S. (2012). An evaluation of carrier screening for spinal muscular atrophy against the National Screening Committee Criteria. UK National Screening Committee. https://legacyscreening.phe.org.uk/policydb_download.php?doc=279. Accessed 18 June 2017.

[CR16] Charmaz K, Holstein JA, Gubrium JF (2008). Constructionism and the grounded theory. Handbook of constructivist research.

[CR17] Chen E, Schiffman J (2000). Attitudes towards genetic counselling and prenatal diagnosis among a group of individuals with physical disabilities. Journal of Genetic Counseling.

[CR18] Cornelis C, Aad T, Dondorp W, Van Haelst M, Bredenoord AL, Knoers N, Düwell M (2016). Whole-exome sequencing in paediatrics- parents’ considerations toward return of unsolicited findings for their child. European Journal of Human Genetics.

[CR19] De Wolfe P (2002). Private tragedy in social context? Disability, illness and suffering. Disability & Society.

[CR20] Dragonas T, Ettore E (2001). Whose fault is it? Shame and guilt for the genetic defect. Before birth: Understanding prenatal screening.

[CR21] Dubowitz V (1991). Chaos in classification of the spinal muscular atrophies of childhood. Neuromuscular Disorders.

[CR22] Edwards SD (2004). Disability, identity and the ‘expressivist objection’. Journal of Medical Ethics.

[CR23] England, G. (2012). *The 100,000 Genomes Project* https://www.genomicsengland.co.uk/the-100000-genomes-project/.

[CR24] Glaser B, Strauss A (1967). The discovery of grounded theory: Strategies for qualitative research.

[CR25] Green JM, Snowdon C, Statham H (1993). Pregnant women’s attitudes to abortion and prenatal screening. Journal of Reproductive and Infant Psychology.

[CR26] Hahn H, Belt T (2004). Disability identity and attitudes towards cure in a sample of disability activists. Journal of Health and Social Behaviour.

[CR27] Hallowell, N., Arden-Jones, A., Eeles, R., Foster, C., Lucassen, A., Moynihan, C. and M. Watson. 2006. ‘Guilt, Blame and Responsibility: Men’s Understanding of their Role in the Transmission of BRCA1/2 Mutations within their Family’ in Sociology of Health and Illness 28 (7) pp. 969–988. 10.1111/j.1467-9566.2006.00515.x 17163862

[CR28] Hauser-Cram P, Erickson Warfield M, Shonkoff JP, Wyngaarden Krauss M, Sayer A, Christofk Upshur C (2001). Children with disabilities: A longitudinal study of child development and parent well-being. Monographs of the Society for Research in Child Development.

[CR29] Ho H, Tseng Y, Hsin Y, Chou F, Lin W (2016). Living with illness and self-transcendence: The lived experience of patients with spinal muscular atrophy. Journal of Advanced Nursing.

[CR30] Jeppesen J, Madsen A, Marquardt J, Rahbek J (2010). Living and ageing with spinal muscular atrophy type 2: Observations among an unexplored patient population. Developmental Neurorehabilitation.

[CR31] Kenen R, Robinson L (1994). The human genome project: Creator of the potentially sick, potentially vulnerable and potentially stigmatised?. Life and death under high technology medicine.

[CR32] Kruitwagen-van Reenen, E., Wadman, R., Visser-Meiley, J., Van der Berg, L., Schröder, C., & Ludo van der Pol, W. (2016). Correlates of health related quality of life in adult patients with spinal muscular atrophy. *Muscle & Nerve*. doi:10.1002/mus.25148. 10.1002/mus.25148 27074445

[CR33] Lamb C, Peden A (2008). Understanding the experience of living with spinal muscular atrophy: A qualitative description. Journal of Neuroscience Nursing.

[CR34] Leo MC, Mcmullen C, Wilfond BS, Lynch FL, Reiss JA, Gilmore MJ, Himes P (2016). Patients' ratings of genetic conditions validate a taxonomy to simplify decisions about preconception carrier screening via genome sequencing. American Journal of Medical Genetics.

[CR35] Locock L, Ziebland S, Dumelow C (2009). Biographical disruption, abruption and repair in the context of motor neurone disease. Sociology of Health & Illness.

[CR36] Malek J (2010). Deciding against disability: Does the use of reproductive genetic technologies express disvalue for people with disabilities?. Journal of Medical Ethics.

[CR37] Maxwell, S. J., Kyne, G., Molster, C., Barker, N. M., Ormsby, J., & O’Leary, P. (2011). Perceptions of population cystic fibrosis prenatal and preconception carrier screening among individuals with cystic fibrosis and their family members. *Genetic Testing and Molecular Biomarkers, 15*(3), 159–164. 10.1089/gtmb.2010.0121 21204697

[CR38] Middleton A, Hewison J, Mueller R (1998). Attitudes of deaf adults towards genetic testing for heredity deafness. American Journal of Human Genetics.

[CR39] Norton M, Nakagawa S, Kuppermann M (2014). Women’s attitudes regarding prenatal testing for a range or congenital disorders of varying severity. Journal of Clinical Medicine.

[CR40] Nuffield Council on Bioethics. (2017). Non-invasive prenatal testing: an ethical review, http://nuffieldbioethics.org/wp-content/uploads/NIPT-ethical-issues-full-report.pdf.

[CR41] Ottesen, E. (2017). ISS-N1 makes the first FDA-approved drug for spinal muscular atrophy. *Translational Neuroscience, 8*(1). doi:10.1515/tnsci-2017-0001. 10.1515/tnsci-2017-0001 PMC5382937 28400976

[CR42] Parens E, Asch A, Parens E, Asch A (2000). The disability rights critique of prenatal testing: Reflections and recommendations. Prenatal testing and disability rights.

[CR43] Pei-Jung L, Wei-Shi Y, Neumann P (2017). Willingness to pay for a newborn screening test for spinal muscular atrophy. Pediatric Neurology.

[CR44] Petersen A (2006). The best experts: The narratives of those who have a genetic condition. Social Science and Medicine.

[CR45] Peterson M (2012). Disability advocacy and reproductive choice: Engaging with the expressivist objection. Journal of Genetic Counseling.

[CR46] Plano Clark VL, Creswell J (2008). The mixed methods reader.

[CR47] Plantinga, M., Birnie, E., Abbott, K., Sinke, R., Lucassen, A., Schuurmans, J. et al. (2016). Population-based preconception carrier screening: How potential users from the general population view a test for 50 serious diseases. *European Journal of human genetics, 24*(10), 1–7. 10.1038/ejhg.2016.43 PMC5027688 27165008

[CR48] Prior TW (2010). Newborn and carrier screening for spinal muscular atrophy. American Journal of Medical Genetics.

[CR49] Pugh C, Healey S, Crane J, Young D (2000). Successful pregnancy and spinal muscular atrophy. Obstetrics and Gynaecology.

[CR50] Reed K (2009). ‘It’s them faulty genes again’: Women, men and the gendered nature of genetic responsibility in prenatal blood screening. Sociology of Health & Illness.

[CR51] Shakespeare, T. (2005). The social context of individual choice, Wasserman, D., Bickenbach, J. Wachbroit, R. (eds) quality of life and human difference: Genetic testing, health care and disability, Cambridge: Cambridge University Press.

[CR52] Shakespeare T (2006). Disability rights and wrongs.

[CR53] Shuttleworth R, Wedgwood N, Wilson N (2012). The dilemma of disabled masculinity. Men and Masculinities.

[CR54] Sinason V (1992). Mental handicap and the human condition.

[CR55] Skinner D, Sparkman KL, Bailey DB (2003). Screening for fragile X syndrome: Parent attitudes and perspectives. Genetics in Medicine.

[CR56] Stern S, Arnos K, Murrelle L, Oelrich Welch K, Nance W, Pandya A (2002). Attitudes of deaf and hard of hearing subjects towards genetic testing and prenatal diagnosis of hearing loss. The Journal of Gene Medicine.

[CR57] Stories, T. (2007). *Understandig real life genetics* http://tellingstories.nhs.uk/.

[CR58] Sukenik-Halevy R, Leil-Zoabi UA, Peled-Perez L, Zlotogora J, Allon-Shalev S (2012). Compliance for genetic screening in the Arab population in Israel. The Israel Medical Association Journal.

[CR59] UK National Screening Committee. (2013). *The UK NSC recommendation on Spinal Muscular Atrophy* https://legacyscreening.phe.org.uk/sma.

[CR60] Von Gontard A, Zerres K, Backes M, Laufersweiler-Plass C, Wendland C, Melchers P, Lehmkuhl G, Rudnik-Schöneborn S (2002). Intelligence and cognitive function in children and adolescents with spinal muscular atrophy. Neuromuscular Disorders.

[CR61] Wang C, Finkel R, Bertini E, Schroth M, Simonds A, Wong B (2007). Consensus statement for standard of care in spinal muscular atrophy. Journal of Child Neurology.

[CR62] Watson N (2002). ‘well I know this is going to sound very strange to you, but I don’t see myself as a disabled person’: Identity and disability. Disability & Society.

[CR63] Watson E, Williamson R, Chapple J (1991). Attitudes to carrier screening for cystic fibrosis: A survey of health care professionals, relatives of sufferers and other members of the public. British Journal of General Practice.

[CR64] Wells D, Kaur K, Griffo J, Glassner M, Taylor JC, Fragouli E, Munne S (2014). Clinical utilisation of a rapid low-pass whole genome sequencing technique for the diagnosis of aneuploidy in human embryos prior to implantation. Journal of Medical Genetics.

[CR65] Willis GB (2005). Cognitive interviewing: A tool for improving questionnaire design.

